# A brief botanical survey into Kumbira forest, an isolated patch of Guineo-Congolian biome

**DOI:** 10.3897/phytokeys.65.8679

**Published:** 2016-06-15

**Authors:** Francisco M. P. Gonçalves, David J. Goyder

**Affiliations:** 1 Herbarium of Lubango, ISCED-Huíla, Sarmento Rodrigues, S/N Lubango, Angola; 2 University of Hamburg, Biocentre Klein Flottbek, Ohnhorststr.18, 22609 Hamburg, Germany; 3 Herbarium, Royal Botanic Gardens, Kew, Richmond, Surrey,TW9 3AB, UK

**Keywords:** Kumbira forest, Guineo-Congolian, floristic diversity

## Abstract

Kumbira forest is a discrete patch of moist forest of Guineo-Congolian biome in Western Angola central scarp and runs through Cuanza Norte and Cuanza Sul province. The project aimed to document the floristic diversity of the Angolan escarpment, a combination of general walk-over survey, plant specimen collection and sight observation was used to aid the characterization of the vegetation. Over 100 plant specimens in flower or fruit were collected within four identified vegetation types. The list of species includes two new records of Guineo-Congolian species in Angola, one new record for the country and one potential new species.

## Introduction

Angola lies almost wholly within the southern zone of tropical grassland, bounded by tropical rain forest of the Congo in the north and by the Kalahari Desert in the south (Shaw 1947). In general the country comprises four main physiographic components: a coastal plain from the Atlantic seaboard of approximately 200 m above sea level, and from 12 to 200 km wide; a narrow steep escarpment, from 200 to 1000 m altitude; an interior plateau, occupying nearly 80% of the country lying between 1000 and 1600 m, and a mountain belt rising above the escarpment and plateau of about 2620 m in central plateau (Huntley and Matos 1994).

Between the Karoo-Namib phytochorion of the coastal belt and the *Brachystegia* dominated Zambesian phytochorion of the interior plateau, a discontinuous series of moister vegetation type extends southwards from the Guinea forest and Congo savanna systems following the escarpment (Huntley 1974, White 1983). The Angolan escarpment is dominated by semi-deciduous forest and a mosaic of forest-savanna and gallery forest of Guineo-Congolian affinity, which is restricted to the interior of Cabinda province and large but discontinuous patches of forest in Zaire, Uíge, Cuanza Norte and Cuanza Sul provinces (Barbosa 1970, Huntley 1974).

These vegetation formations, as referred above, cover large areas of Cabinda with a tree strata of about 30 to 40 m height, while in the south are restricted to extensive “Coffee forests” in Dembos, Cazengo and Gabela regions (Huntley 1974). In this latter Kumbira forest, located in Conda, Cuanza Sul province is no doubt the most important and, probably the most southerly and most isolated patch of this biome, with various elements of Congo basin and West African affinities, dominated by genera *Celtis*, *Morus*, *Albizia*, *Bombax* and *Pterocarpus* (Barbosa 1970).

To these formations can be added the afro-montane forests, also of great biogeographic interest (Hall 1960a), restricted to small and isolated patches of forest in Benguela, Huambo and Huíla provinces. The total area of these forest patches was estimated to be approximately 200 ha, the best known of which is Mount Moco in Huambo province which provides habitat for its flora and avifauna (Huntley 1974, Olmos 2008 unpub., Maiato 2009, Mills 2010). The Angolan escarpment with approximately 1000 km of extent is unique, beautiful and constitutes the section of the Great Escarpment of Southern Africa poorly known in terms of its biodiversity, but high level of endemism (Huntley and Matos 1994, Figueiredo 2008, Clark 2011).

Despite the recent published checklist of Angolan vascular plants (Figueiredo and Smith 2008), data on the Angolan flora is mostly limited to the literature of pre-independence era, and current knowledge on Angolan plants is poor and restricted to isolated and focused studies carried out by individuals or institutions. Increasing interest and efforts are being made in order to document and obtain baseline data of areas with high socio-ecological importance (for example The Future Okavango Project – http://www.future-okavango.org and the Okavango Wilderness Project – http://www.wildbirdtrust.com/portfolio/okavango-widerness-project) which focus on the Cubango and Cuito river catchments respectively). However large parts of the country, such as the Angolan escarpment, remain to be studied biologically.

In terms of biodiversity, only the avifauna has been investigated in any detail (Hall 1960a, Cagan and Riley 2005, Mills 2010, Cáceres et al. 2015). Recommendations have being made to undertake botanical surveys in the Kumbira area (Barbosa 1970, Huntley 1974). The ecological importance of Kumbira forest in maintaining the highest number of Angolan endemic avifauna highlighted the area as important for conservation of biodiversity. But at the same time concerned due to the increasing human pressure (Huntley 2011 unpub.). To fill in the gap in terms of botanical diversity, we undertook a botanical survey into Kumbira forest. Here we document our current knowledge of the plant diversity and phytogeographical affinities of Kumbira Forest, the findings are based primarily on a rapid botanical assessment conducted between 10^th^ and 18^th^ June 2014.

## Methods

### Study area

**Figure 1.**
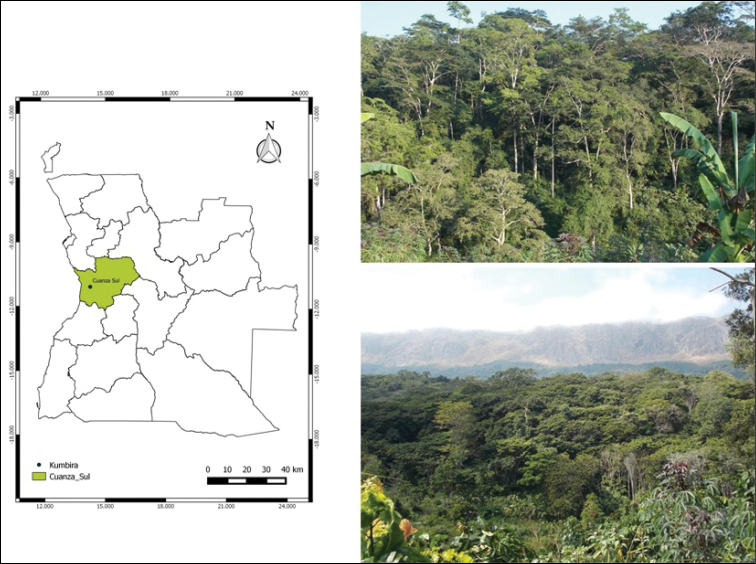
Map of Angola and its provinces, Cuanza Sul province highlighted and Kumbira in Conda municipality (black dot). Kumbira forest (top), the forest with Njelo mountain in the background (bottom).

The central escarpment of western Angola on which Kumbira forest is part of, runs through Cuanza Norte and Cuanza Sul provinces. Barbosa (1970) recognized three types of moist high forest along this escarpment occurring in discrete geographic and climatic zones. North to south, these are:

i. forests of subtype Cazengo to the north of the Cuanza River;

ii. forests of subtype Amboim, between the Cuanza and Keve (Queve) Rivers, of which the most significant is the area of forest around Gabela;

iii. forests of subtype Uku (Vila Nova do Seles) to the south of the Keve River;

Kumbira forest (11°07.00'S; 014°17.00'E) is a discrete patch of moist forest vegetation in this third zone, SW of Conda (Figure 1), where cloud gathers under the knife-edge ridge of the Njelo mountain which reaches around 1500 m in elevation and prevents the cloud from moving further inland. The forest forms part of about 200 000 ha of semi-deciduous moist forest (Cagan and Riley 2005) and occurs on middle altitude slopes at around 700–900 m. Below this altitude dry open vegetation predominates, and at higher elevation the forest gives way to woodland and ultimately open rocky mountain slopes into rocky gorges in Njelo mountain.

Kumbira forest is considered to be part of Gabela Important Bird Area (Dean 2001), and holds unique and threatened bird community (Cagan and Riley 2005). Although its ornithological importance the area is not protected by law, the proposed implementation of Gabela Natural Reserve, recently reinforced (Huntley 1974; Huntley 2011 unpub.) is unfortunately far from being achieved.

### Sampling strategy

The botanical team consisted of staff from the Royal Botanic Gardens Kew, UK and from the Herbarium of Lubango, Angola. The team used a combination of general walk-over survey (Filgueiras et al. 1994), plant specimen collection and sight observations to aid the characterization of the vegetation and the compiling of an inventory of the plant species present. Over 100 herbarium specimens were collected, mostly of flowering and fruiting plants, but some sterile collections (lacking flowers or fruits) were made of the more common or important species which were not flowering or fruiting at the time of our visit.

Due to the limited time available, no plot-based surveys to quantify the composition of the different vegetation types were conducted. Plant specimens were collected in duplicate or triplicate, with one set deposited in Lubango for incorporation into the main collection, the remaining set(s) being taken back to the UK where they were identified by comparison with material housed in the Kew herbarium. A range of botanical specialists were consulted to help in the identification of difficult plant groups.

Due to the paucity of useful literature on Angolan plants, and to the limited previous botanical work in the region, it was not possible to name all collections made, particularly sterile material, but we were able to name the large majority. Those named only to genus are nevertheless included within the checklist in Appendix 1. We have not included the additional species listed by Gossweiler and Mendonça (1939) and Barbosa (1970) from this forest subtype in the Appendix 1, as it is not possible to say which forest patch they are from.

The recent Angolan plant checklist by Figueiredo and Smith (2008) was used as the baseline for assessing species records and distributions in the country. The African Plants Database (http://www.ville-ge.ch/musinfo/bd/cjb/africa/recherche.php) and the World Checklist series (http://apps.kew.org/wcsp/home.do) for a number of plant families were used as the standards for up-to-date taxonomy of African plant species.

## Results

From the vegetation survey carried out in Kumbira forest, four main vegetation types were identified. Here we provide a very brief overview of the forest types and their dominant species as recorded by the rapid botanical survey. The habitat types outlined here are also assigned to each of the species listed in (Appendix 1).

• **Moist high forest (F)**

Surveyed at former coffee plantation at foot of Serra Njelo, c.7 km SW of Conda [centred on 11°09.26'S, 014°17.56'E]). The canopy trees in this part of the forest were retained as shade for the coffee grown underneath (*Coffea canephora*, *Coffea robusta*), and there has been considerable regrowth of forest understorey since the plantations were abandoned. The canopy is c. 25-30 m high, and the commonest tree is the seasonally deciduous *Albizia adianthifolia*, *Trema orientalis*, *Markhamia zanzibarica*, *Antidesma venosum* and several species of *Ficus* are common elements. We also encountered *Anthocleista schweinfurthii*, *Cola welwitschii*, *Pteleopsis diptera*, *Synsepalum cerasiferum*, *Turraea vogelii* and *Vitex welwitschii* in some areas. An arborescent *Dracaena* and a species of *Erythrina* were also noted (sight records only).The understorey was rich in Rubiaceae, and the herbaceous flora included many ferns and occasional epiphytes, *Justicia paxiana* is recorded from Angola for the first time.

**Figure 2.**
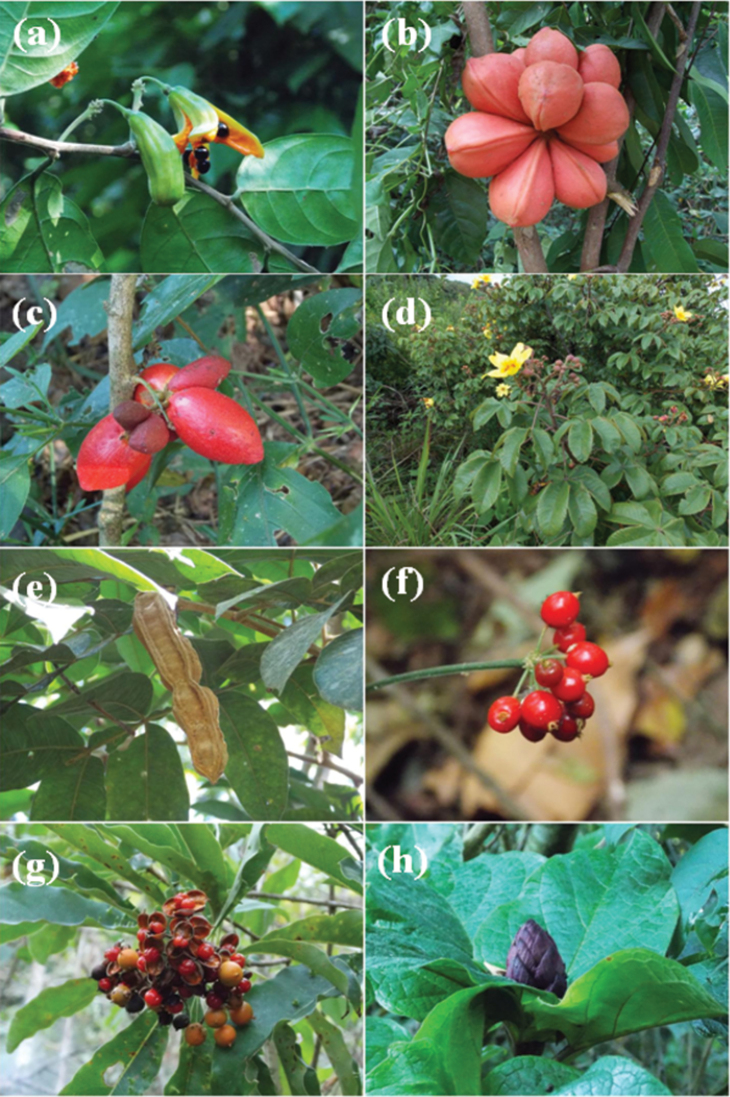
*Turraea vogelii* Hook.f. ex Benth, *Cola welwitschii* Exell & Mendonça ex R. Germ., *Pancovia golungensis* (Hiern) Exell & Mendonça, *Cochlospermum angolense* Welw. ex Oliv., Inga vera Willd. subsp. vera, *Pavetta gossweileri* Bremek, *Pittosporum viridiflorum* Sims, *Clerodendrum poggei* Gürke

• **Submontane forest/woodland (W)**

(Upper margins of Moist High Forest). The moist high forest gives way to deciduous woodland at higher elevations (above c. 900 m). Characteristic trees and shrubs include *Harungana madagascariensis*, *Dombeya rotundifolia*, *Cochlospermum angolense*, *Grewia flavescens*, *Pittosporum viridiflorum* and *Hymenocardia acida*. We also encountered *Podocarpus milanjianus*, an afromontane element, in the gullies at high altitude.

• ***Inga*-dominated former coffee plantation (*Inga*)**

(Surveyed at: mostly at Monte Belo estate, former coffee plantation at foot of Serra Njelo, c. 11 km SW of Conda [centred on 11°10.68'S, 014°16.36'E]). The exotic, evergreen *Inga* trees (*Inga vera* from northern South America) planted initially as shade for the coffee are now overgrown and let little light through the canopy, seriously reducing the development of a shrub or herb layer except in gaps. *Inga vera* was recently referred as one of the most impressive and worrying invasive species of western Angola, forming dense stands in localized sites as observed in Kumbira forest (Rajmánek et al. 2016). Nevertheless, we did record a variety of ferns and other herbaceous plants in this area, in addition to the very large fig *Ficus saussureana*, the first record of this Guineo-Congolian species from Angola. We observed some regeneration of *Inga* from seed, demonstrating its potential as an invasive species. A second exotic mimosoid legume tree was encountered in this area – *Leucaena leucocephala* – also native to the New World.

• **Ruderal and secondary habitats (Sec)**

There is extensive encroachment of agriculture in the vicinity of villages, roads and tracks, and extraction of timber trees in some areas of forest. We did not survey this beyond making occasional observations. The commonest subsistence crops grown in the region are manioc (cassava) and plantain. The oil palm *Elaeis guineensis* is widely planted. We also encountered dense stands of the pigeon pea *Cajanus cajan* in one area. Disturbed areas of forest, recently cleared, had large patches of the invasive shrub *Solanum mauritianum*.

• **Plant diversity**

Our records are a brief snapshot, based on a visit of just six collecting days, at just one time of the year (June). They are by no means comprehensive. Nevertheless, we recorded 92 species from the forest.

• **New plant records for Angola**

We report two new records of Guineo-Congolian forest species for Angola – the tree *Ficus saussureana* and the herb *Justicia paxiana*. In addition, *Tarenna pavettoides* is newly recorded for the country, and may represent an undescribed subspecies. We also report a potential new species in *Rytigynia* sp. nov. (Appendix 2).

## Discussion

### Phytogeographical context

Coastal regions of Angola are strongly influenced by the Benguela cold-water current which reduces surface evaporation and rainfall. Although the current moves off-shore north of Namibe, corresponding with the northern limit of the Namib Desert, coastal vegetation north of the desert remains dry, and is dominated by dry scrub woodland and succulents such as aloes, euphorbias and baobabs.

Kumbira Forest is a very localized patch of forest between this dry coastal vegetation and the moist savannas of the plateau. Barbosa (1970) regarded the forest as typically Zambezian, the canopy dominated by *Albizia* species that lose their leaves in the dry season. Barbosa also quoted from Gossweiler and Mendonça (1939) who commented on the high percentage of *Ficus* species. However, we found that most of the species within the forest are Guineo-Congolian rather that Zambezian in floristic affinity *sensu* White (1983) - the species recorded are generally widespread within the Guineo-Congolian phytogeographical region but often rare or previously unrecorded in Angola.

And it must be noted that *Albizia adianthifolia* which we encountered in the forest, is distributed widely across tropical Africa, and not just in the Zambezian region. Kumbira Forest must be one of the most southerly areas in western Angola with significant Guineo-Congolian vegetation. The upper limits of the forest contain occasional afromontane elements, and the forest merges into moist woodland characterized by widespread species.

## Conclusions

We surveyed key sites in a small portion of Kumbira Forest in Cuanza Sul Province, reporting on diversity and floristic affinities of the flora. The vegetation types were identified: moist high forest, submontane forest & woodland, and *Inga*-dominated former coffee plantation. In addition, ruderal or disturbed areas also occur.

Species composition is overwhelmingly Guineo-Congolian, and this forest represents one of the most southerly areas of such vegetation along the Angolan escarpment. Most species have a wide geographic distribution, but some such as *Pavetta gossweileri* are more restricted, and are reported from just Cuanza Sul and Cuanza Norte. *Deinbollia laurifolia* is restricted to riverine lowland habitats from Cuanza Sul to Cameroon.

Much of the area surveyed had good regeneration of the understorey since coffee cultivation ceased, but we observed large trees being taken out of the forest, and evidence of village agriculture encroaching in a number of places. Areas formerly planted with New World legume species, particularly *Inga vera*, were in less favourable condition as the overgrown *Inga* shades out the understorey. There is some evidence of this species reproducing and spreading naturally, and we observed other potentially invasive species such as *Solanum mauritianum*. In addition, some coffee estates are being reestablished, which will reduce the extent of undisturbed or recovering forest further. Three species are newly recorded for Angola, *Ficus saussureana*, *Justicia paxiana* and *Tarenna pavettoides*. We also report a potential new species of *Rytigynia*.

## Appendix 1

**Table:** List of the vascular plants collected and observed in Kumbira Forest, Cuanza Sul, Angola. Habitat types in Kumbira forest relate to the phytochoria recognised by White (1983) as follows: Forest = I. Guineo-Congolian; Woodland = VIII. Afromontane. Most species found in secondary forest and *Inga*-dominated forest are also Guineo-Congolian in distribution and affinity.

**Family**	**Species**	**Habitat type**	**Collectors**	**Voucher number(s)**
**PTERIDOPHYTA**				
**Dennstaedtiaceae**	*Blotiella currorii* (Hook.) R.M.Tryon	Forest	Goyder & Maiato	7701, 7719, 7720, 7722
**Dryopteridaceae**	*Bolbitis gemmifera* (Hieron.) C.Chr.	Forest	Goyder & Maiato	7702, 7726
**Lomariopsidaceae**	cf. *Lomariopsis*	Forest	Goyder & Maiato	7718
**Polypodiaceae**	*Platycerium* sp.	Forest	Sight record	
**Pteridaceae**	Pteris catoptera Kunze var. catoptera	Inga	Goyder & Maiato	7783
**Thelypteridaceae**	*Christella dentata* (Forssk.) Brownsey & Jermy	Forest	Goyder & Maiato	7721, 7727
**GYMNOSPERMAE**				
**Podocarpaceae**	*Podocarpus milanjianus* Rendle	Woodland	Goyder & Maiato	77800
**ANGIOSPERMAE**				
**Araceae**	*Culcasia angolensis* Welw. ex Scott	Forest	Goyder & Maiato	7802
**Asparagaceae**	Asparagus africanus Lam. var. puberulus (Baker) Sebseb	Forest	Goyder & Maiato	7751
	*Dracaena* sp.	Forest	Sight record	
**Commelinaceae**	*Aneilema beniniense* (P.Beauv.) Kunth	Forest	Goyder & Maiato	7741
	Palisota cf. schweinfurthii C.B.Clarke	Forest	Goyder & Maiato	7715
**Dioscoreaceae**	*Dioscorea praehensilis* Benth.	Forest	Goyder & Maiato	7758
**Marantaceae**	*Marantochloa leucantha* (K.Schum.) Milne-Rendh.	Forest	Goyder & Maiato	7778
**Acanthaceae**	*Acanthus montanus* (Nees) T.Anderson	Forest	Goyder & Maiato	7707
	*Justicia flava* (Vahl) Vahl	Secondary forest	Sight record	
	*Justicia paxiana* Lindau	Forest	Goyder & Maiato	7717
**Apocynaceae**	*Motandra guineensis* (Thonn.) A.DC	Forest	Goyder & Maiato	7804
	*Oncinotis* sp.	Forest	Goyder & Maiato	7777
**Bignoniaceae**	*Markhamia zanzibarica* (Bojer ex DC.) K.Schum.	Forest	Goyder & Maiato	7710
**Bixaceae**	*Cochlospermum angolense* Welw. ex Oliv.	Woodland	Goyder & Maiato	7796
**Cactaceae**	*Rhipsalis baccifera* (J.S.Muell.) Stearn	Forest/Inga	Sight record	
**Cannabaceae**	*Trema orientalis* (L.) Blume	Forest	Goyder & Maiato	7765
**Cleomaceae**	*Cleome* sp.	Forest	Goyder & Maiato	7713
**Combretaceae**	*Combretum angolense* Welw. ex M.A.Lawson	Forest	Goyder & Maiato	7712
	*Combretum collinum* Fresen.	Woodland	Goyder & Maiato	7792
	*Combretum platypetalum* Welw. ex M.A.Lawson	Forest	Goyder & Maiato	7757, 7766
	*Pteleopsis diptera* Engl. & Diels	Forest/woodland	Goyder & Maiato	7725, 7793
**Connaraceae**	*Rourea thomsonii* (Baker) Jongkind	Forest	Goyder & Maiato	7703
**Dichapetalaceae**	*Dichapetalum crassifolium* Chodat	Forest	Goyder & Maiato	7704
**Ebenaceae**	*Diospyros heterotricha* (Welw. ex Hiern) F.White	Forest	Goyder & Maiato	7704
**Euphorbiaceae**	*Acalypha paniculata* Miq.	Forest	Goyder & Maiato	7762
	*Antidesma venosum* E.Mey. ex Tul	Forest	Goyder & Maiato	7768
	*Croton gratissimus* Burch.	Forest	Goyder & Maiato	7801
	*Mallotus oppositifolius* (Geiseler) Müll. Arg.	Forest	Goyder & Maiato	7734
**Gentianaceae**	*Anthocleista schweinfurthii* Gilg.	Forest	Goyder & Maiato	7774
**Hypericaceae**	*Harungana madagascariensis* Lam. ex Poir.	Woodland	Goyder & Maiato	7791
**Lamiaceaee**	*Clerodendrum poggei* Gürke	Forest	Goyder & Maiato	7736
	*Clerodendrum volubile* P.Beauv.	Forest	Goyder & Maiato	7714
	*Vitex welwitschii* Gürke	Forest	Goyder & Maiato	7770
**Leguminosae: Mimosoideae**	*Acacia pentagona* (Schumach. & Thonn.) Hook.f.	Forest	Goyder & Maiato	7748
	*Albizia adianthifolia* (Schumach.) W.Wight	Forest	Goyder & Maiato	7740, 7764
	Inga vera Willd. subsp. vera	Inga	Goyder & Maiato	7759, 7764
	*Leucaena leucocephala* (Lam.) de Wit	Inga	Goyder & Maiato	7780
**Leguminosae: Papilionoideae**	*Cajanus cajan* (L.) Millsp.	Secondary forest	Goyder & Maiato	7784
	*Dalbergia saxatilis* Hook.f.var. saxatilis	Forest	Goyder & Maiato	7769
	*Desmodium repandum* (Vahl) DC	Forest	Goyder & Maiato	7742
	*Erythrina* sp.	Forest	Sight record	
	*Millettia drastica* Welw. ex Baker	Forest	Goyder & Maiato	7787
	*Neonotonia wightii* (Widht & Arn.) J.A.Lackey	Forest	Goyder & Maiato	7723
	*Psophocarpus scandens* (Engl.) Verdc.	Secondary forest	Goyder & Maiato	7785
**Loranthaceae**	*Tapinanthus constrictiflorus* (Engl.) Danser	Forest	Goyder & Maiato	7789
**Malvaceae**	*Cola welwitschii* Exell & Mendonça ex R. Germ.	Forest	Goyder & Maiato	7730, 7747
	Dombeya rotundifolia (Hochst.) Planch. var. rotundifolia	Woodland	Goyder & Maiato	7795
	*Grewia flavescens* Juss.	Woodland	Goyder & Maiato	7794
	*Grewia floribunda* Mast.	Forest	Goyder & Maiato	7744
	*Urena lobata* L.	Secondary forest	Goyder & Maiato	7776
**Meliaceae**	*Turraea vogelii* Hook.f.ex Benth	Forest	Goyder & Maiato	7728, 7756
**Moraceae**	*Ficus conraui* Warb.	Inga	Goyder & Maiato	7761
	*Ficus saussureana* DC.	Inga	Goyder & Maiato	7782
	*Ficus* sp.	Forest	Goyder & Maiato	7739
	*Trilepisium madagascariense* DC.	Forest	Goyder & Maiato	7733
**Passifloraceae**	Adenia lobata (Jacq.) Engl. subsp. lobata	Forest	Goyder & Maiato	7752
**Phyllanthaceae**	*Hymenocardia acida* Tul.	Woodland	Goyder & Maiato	7798
	*Thecacoris trichogyne* Müll. Arg.	Forest	Goyder & Maiato	7716
**Pittosporaceae**	*Pittosporum viridiflorum* Sims	Woodland	Goyder & Maiato	7797
**Primulaceae**	*Maesa welwitschii* Gilg.	Forest	Goyder & Maiato	7709
**Rhamnaceae**	*Gouania longipetala* Hemsl.	Forest	Goyder & Maiato	7724
**Rubiaceae**	*Bertiera orthopetala* (Hiern) N.Hallé	Forest	Goyder & Maiato	7771
	*Coffea canephora* Pierre ex A.Froehner	Forest/Inga	Goyder & Maiato	7786
	*Empogona glabra* (K.Schum.) Tosh & Robbr.	Forest/Inga	Goyder & Maiato	7755
	*Mussaenda erythrophylla* Schumach. & Thonn.	Forest	Goyder & Maiato	7803
	*Pavetta gossweileri* Bremek	Forest/Inga	Goyder & Maiato	7760
	*Psychotria nigropunctata* Hiern	Woodland	Goyder & Maiato	7799
	*Rothmannia longiflora* Salisb.	Forest	Goyder & Maiato	7745
	*Rothmannia whitfieldii* (Lindl.) Dandy	Forest	Goyder & Maiato	7746
	*Sherbournia bignoniiflora* (Welw.) Hua	Forest	Goyder & Maiato	7705
	*Tarenna pavettoides* (Harv.) Sim?subsp. nov.	Forest/Inga	Goyder & Maiato	7775
	*Rytigynia*?sp. nov.	Forest	Goyder & Maiato	7788
**Sapindaceae**	*Deinbollia laurifolia* Baker	Forest	Goyder & Maiato	7743
	*Pancovia golungensis* (Hiern) Exell & Mendonça	Forest	Goyder & Maiato	7772
	*Paullinia pinnata* L.	Forest	Goyder & Maiato	7767
**Sapotaceae**	*Synsepalum cerasiferum* (Welw.) T.D.Penn.	Forest	Goyder & Maiato	7754
**Solanaceae**	*Solanum anomalum* Thonn.	Forest	Goyder & Maiato	7711
	*Solanum mauritianum* Scop.	Secondary forest	Goyder & Maiato	7738
	*Solanum terminale* Forssk.	Forest	Goyder & Maiato	7749
**Umbelliferae**	*Steganotaenia araliacea* Hochst.	Inga	Goyder & Maiato	7779
**Urticaceae**	*Urera trinervis* (Hochst.) Friis & Immelman	Forest	Goyder & Maiato	7773
**Violaceae**	*Rinorea ilicifolia* (Welw. ex Oliv.) Kuntze	Forest	Goyder & Maiato	7750
**Vitaceae**	*Cissus aralioides* (Welw. ex Baker) Planch.	Forest	Goyder & Maiato	7790
	*Leea guineensis* G.Don	Secondary forest	Goyder & Maiato	7708

## Appendix 2

**Table:** List of species collected in Kumbira forest which represent new records for Angola.

**Family**	**Species**	**Notes**
**Acanthaceae**	*Justicia paxiana* Lindau	Widely distributed in West Africa and the Congo Basin, but not recorded before from Angola
**Moraceae**	*Ficus saussureana* DC.	Widely distributed in West Africa and eastern and western margins of the Congo Basin, but not recorded before in Angola.
**Rubiaceae**	*Rytigynia* ?sp. nov.	We were unable to match this to any known species of the genus.
**Rubiaceae**	*Tarenna pavettoides* (Harv.) Sim	This species has one subspecies distributed across West Africa and northern limits of the Congo Basin, and other subspecies in Eastern and Southern Africa. It has not been reported from Angola before, and our collection may represent a new subspecies.
